# Resignation

**Published:** 1869-05

**Authors:** 


### RESIGNATION.

We knew a man in our happy youth-time, who seemed to us to be all sunshine; kind and cordial to everybody; as expressive of the general feeling towards him, he was known by the name of "Uncle Davy." He was a great friend to the church and the "preachers." We used to be at his house, sometimes every day, at others at long intervals, and was always received with not a seeming, but a real welcome; a generation has passed since then, but it would be the same to-morrow. When the church needed money for any good thing, he was not the man to refuse his mite. Men of character are very apt to have "crotchets;" some striking likes or dislikes. It was a little thing, but it seemed to be done with such a hearty good will, such genuine courtesy, that it has never been forgotten. He belonged to the "McChord" church, the pews were so arranged that they faced the doors, for the benevolent purpose of preventing persons from twisting their necks off, in turning round to look at the pretty girls and others who came in late, and also to compel all to remain in church until the services were over, as any one leaving would have to march down the aisle in the very face of the minister, who made it a point when he saw one coming to stop instantly until the abashed individual, in awful silence, had closed the door after him; no man was ever known to try that feat twice. Uncle Davy owned the front pew for himself and good wife; but the very moment a stranger entered the door, he was on his feet in the centre of the aisle bowing the visitor into his pew; when that was full he would in the same way fill up his other pew; this was a true welcome to the house of God, and falls sweetly on the mind of the transient comer; it soothes and fits it for the reception of the preached word which is sown then like good seed on a prepared soil.

One day the "Session" took it into their heads that it would be better for the church to educate its own daughters, and that it would be a great thing if they had a "session room," which could be also used for a Sabbath school, for prayer meetings, and an academy for young ladies. Well, they talked it over, and asked Uncle Davy about it. "Thirtingly," said he, "it's a first rate plan." That was the end of it! But not the everlasting end; for not a great while after the land was bought and the house built, and the deed handed to the church at a cost of Thirty Thousand Dollars, and every dollar out of "Uncle Davy's" pocket; and other like things, and better things, and greater things, he has been doing from time to time ever since. But in the mean time Uncle Davy has got to be an old man in body, but as young in feeling as he ever was. But we all do fade as a leaf. The other day he wrote, "Found me confined to a sick room with my old complaint, the rheumatism, and my good wife is confined to the house much of the time from the same disease; but we try to be resigned to God's providence, and submit with cheerfulness to his righteous will. Although we suffer much pain, we try to be cheerful and love everybody, and do them good, and give our hearts to Jesus Christ, and that makes all men happy. I am now seventy-six years old, and am pretty well worn out."

Here is a long life of active benevolence, closing with a happy confidence in the dispensations of Providence, an abiding feeling that "all is well." Reader, may you and I so live henceforth, that when we come in sight of "fourscore," we may have, well grounded, the hope and the faith of "Uncle Davy."

